# Dental Society of the State of New York

**Published:** 1885-06

**Authors:** 


### DENTAL SOCIETY OF THE STATE OF NEW YORK.

### SEVENTEENTH ANNUAL MEETING, HELD AT ALBANY, MAY, 1885.

The Dental Society of the State of New York convened for its seventeenth annual meeting at Agricultural Hall, in the State Geological Building, at Albany, on May 13th, 1885.

The regular annual meeting of the Board of State Censors, for the examination of candidates, had been held on the day preceding. Twelve had presented themselves for examination, of whom three were recommended to the Society for its diploma.

A large number of permanent and delegate members were present, with a number of visitors, among whom was W. H. Waite, D. D. S., of Liverpool, Eng., an honorary member of the society.

After the usual routine business, and the presentation of reports from each of the eight District Societies, and the Colleges of the the State, the President, Dr. L. S. Straw, delivered his annual address.

The Corresponding Secretary, Dr. W. H. Atkinson, then read his report, which was objected to by some of the members, as possessing more of the character of an essay than a report. In reply, the doctor stated that he had communicated with various societies and members of societies, and that the replies received touched more upon local matters than upon scientific subjects, and that he had prepared this paper in place of a "report."

It was moved and carried that his verbal answer be incorporated as the report, and the paper referred to the publication committee.

The degree of Master of Dental Surgery was conferred upon the following gentlemen, whom the Censors reported as having passed a satisfactory examination:

Charles F. Ives, New York; B. A. R. Ottolenguin, Brooklyn; E. Judson Taylor, Syracuse.

Other routine business then followed, after which the Committee on Codification of the Dental Laws reported through its chairman, Dr. N. W. Kingsley.

This report was the subject of much discussion, several of the members being of the opinion that the old laws should remain as they were, and that only such as were new should be adopted by the Society.

Dr. Kingsley stated that the object of codification was to bring all the laws of the Society into a convenient form. That, previous to this, they had existed as by-laws, standing resolutions, etc., and that if the old laws were left and the new portions adopted, it would practically defeat the object for which the committee had been appointed, and to the furtherance of which they had devoted much time and labor.

Dr. Barrett moved the adoption of the report, except the appendix, and subsequently desired to add to his motion that the report be referred back to the committee, with instructions to also prepare a digest of the dental laws of the State.

Upon a division of the question the latter part of Dr. Barrett's motion was lost, and the original motion, for the adoption of the report, except the appendix, was carried.

After further routine business the Society took a recess till 3.30 P. M.

AFTERNOON SESSION.

The President called the meeting to order, and after the minutes of the morning session were read the privileges of the floor were, upon motion of Dr. Kingsley, extended to all gentlemen present who were connected with dental colleges, and to all visiting dentists.

Routine business was then proceeded with, after which "Incidents of Office Practice" was called up:

*Dr. Jarvie*—I have a case that I would like to present to the attention of the Society. A gentleman, in August last, fell and broke the point off the upper superior central incisor, and was very averse to having it built up with gold. He preferred to have the tooth remain as it was, rather than have it disfigured with a lump of gold. He was about thirty-five years of age. I ground a piece of porcelain, as like the tooth in color as I could select, and had a hole drilled in it as large as it would permit, without danger of fracture, and a corresponding hole made in the natural tooth, and cemented it on. The model is here, showing the natural teeth before I did anything, and a model as I took it on Saturday. I performed the operation, I think, last September, and they are together to-day.

In response to questions, the doctor stated that the pulps were alive, and that the cement he used was oxy-phosphate of zinc.

*Dr. Frank Abbott*—I recently had a similar case, a lower incisor. The difficulty in proceeding as did Dr. Jarvie, was that the tooth was dead. I took a piece of a porcelain tooth, the color that I wanted, and had it ground carefully to fit the tooth, so that the joint would be almost absolute, carefully preserving the platinum pins in the center. This I backed up with gold, laying the gold down behind and over the capping, and over the end of the tooth, and from that a piece of wire was carried into the root of the tooth, to hold it, in the way Dr. Jarvie speaks of, except that I ground off the tooth where the points were, and put gold behind. I have put pieces on in that way several times.

*Dr. Rich*—Do such teeth stand well and permanently?

*Dr. Abbott*—The first one that I ever fixed in this way was about fifteen years ago. After six or eight months the young man went to Europe, and I have never seen him since. I have never had good success in capping with pieces of teeth in the way of which Dr. Jarvie speaks, unless the piece was very large. They do not stand as a piece will that has a metal backing, and the patient can bite upon the metal. I have gone so far as to take a piece of platinum and fit it over the end of a tooth as perfectly as I could, and then bake a regular enamel upon it.

The best success I have ever had in work of this kind, was by driving a piece of dry hickory wood into a hole drilled in the tooth, and into this driving a piece of gold wire. Upon this can be built a cement that shall restore the appearance of the tooth.

*Dr. Jarvie*—There is another case that I wish to report, on account of what at the time seemed a little peculiar. A boy, about ten years of age, fell on the ice and knocked out the two superior incisors. They remained in the yard for some time, were then found and brought into the house. The outer plate of the alveolus was broken, the teeth themselves broken, the cutting edges broken. I ground the crowns of the teeth into shape, and cut off the extreme end of the root. I removed the pulps and filled the roots with oxychloride of zinc and gutta percha. Before putting them back, an impression was taken and a plate made, covering the first permanent and the temporary molars, and the teeth were put back into place, the plate holding them so firmly in position that no motion was perceptible. The teeth became so firmly fixed in place that you could not produce the slightest movement, as though a union had taken place between the socket and the root. The lateral incisors had considerable movement upon pressure, the periosteum yielding to pressure upon it, but with the centrals there seemed to be no movement whatever. I sent the boy to Dr. Mirick and to Dr. Hill, so that the case could be fully verified.

*Dr. Mirick*—I can corroborate all that Dr. Jarvie has said about these teeth. The success was all that any one could have wished, and the teeth were firmer than I would expect in a boy of that age.

Dr. Jarvie wished me to see if there was any osseous deposit. I think the pressure of the alveolus bound the teeth firmly in the sockets.

There may be some gentleman here who was present at a meeting and dinner of the Second District Dental Society, about fifteen years ago. At that meeting I presented a patient who had a tooth knocked out in Wall Street, a young man of about twenty years of age. He had scraped the tooth and made it perfectly clean. He met with the accident about four in the afternoon, and about nine in the evening he presented himself to me, and I returned it to the socket and tied it with ligatures. That tooth became so firm that at this meeting, where I had him on exhibition, no one was able to detect which tooth had been removed. Every one selected the other tooth. The one I had put back was the firmest, and no difference in color could be detected. Within the last year a relative of this young man told me that the tooth had dropped out, as he expressed it.

*Dr. Rhein*—A young lady presented herself to me, one of whose central incisors had been knocked out and was hanging from the mouth by a shred of the tissue. It was replaced, and when examined two months ago, by aid of the electric light, the pulp was alive. It was as firm as the other, although the accident occurred in 1876 or 1877.

He also referred to the case of his brother, who had knocked out two central incisors playing base ball. They were carefully removed and filled, and the extreme point of the root removed, when they were replaced. The result was very similar to the case reported by Dr. Mirick, for about two years ago they both fell out, although for three or four years they were very firm.

The resolution passed last year, requesting the Baltimore College of Dental Surgery to show cause why its name should not be stricken from the list of colleges recognized by the Dental Society of the State of New York, for alleged irregular granting of degrees, was called up, when Prof. R. B. Winder, the Dean, appeared in answer. He said that it was very embarrassing for him to appear before the Society, because no formal charges had been made. The College had simply been cited to appear and show cause why its name should not be stricken from the list of colleges recognized by this Society. He thought great injustice had been done the College, because it had been pilloried for a year, with no chance to make reply. It had been reproached as being under the ban of the Society of the State of New York, when nothing but *ex parte* proceedings had been held. He might plead many things, but he would not affect ignorance of the objections raised. By its charter the College was bound to recognize merit wherever it met it. He himself believed in granting diplomas by merit, and not according to any fanciful or arbitrary standard of time. The Baltimore College has always granted degrees at the close of a successful examination, no matter whether the student had attended lectures or not. That has been the custom of the school for a period at least thirty years longer than the whole age of this Society. It is a regulation included in its charter. To-day, if any man who has been in practice for ten years should present himself and demand an examination, they could not refuse him. They had consulted counsel, and had been told that, as this was in their charter, they could not avoid granting degrees in such cases, in any other way than by raising the standard of graduation quite out of reach. Certainly it was a principle that was amply sustained by the Dental Society of the State of New York, for he had that day witnessed the conferring of a degree by its President, upon a mere examination by the Board of State Censors.

In 1881 Prof. Taft called a convention of the Deans of the Colleges at Niagara, and a National Association was formed, and it was agreed not to recognize the diploma of any school that did not demand attendance upon two full courses of lectures, as an essential to graduation. Baltimore opposed this, because they did not believe that the time qualification was the proper one upon which to issue diplomas. But we could not afford to have our diplomas tabooed. No school can afford to be placed in the position of having its honors dishonored. Besides, I for one believe in submitting to the decision of the majority. The late war taught me that, and so we have signed the agreement, and henceforth shall be bound by the rules of the National Board. I told the gentlemen composing the Board that I did not believe in the graduation of students by the time plan, regardless of merit, but as I was the only one present who advocated other views, and as I did not desire to be an obstructionist, we would abide by the decision of the majority, and the College has done as it pledged itself to do. There will be another meeting this summer, at Chicago, and at that meeting definite plans for the future will be presented.

*Dr. Barrett*—Said that he had advocated the resolution of this Society, passed a year ago, but he had not done it in hostility to the Baltimore, or any other special school. He could now see that an injury had been done the Baltimore College, but it was one that was not intended, nor recognized at the time. The wrong was in giving it no chance to make reply until a year had passed. Every accused person or institution has the right to a speedy trial, and when charges were allowed to be bandied about for a year, to the prejudice of a school, without its having an opportunity to make reply, it was an injustice which he was sorry had been committed.

But this was a question of principles, and not men. Degrees had been loosely granted by more than one American college, and as a consequence American dentistry, and American schools, and the distinctive American degree, had been brought to open shame. England had practically closed her doors against our degree. Germany refused to acknowledge it. France was moving to shut out American dentists, and even Switzerland was legislating against it. Whereas, once the name of American dentist and graduate of an American dental college was the proudest title that one could bear, and was a distinction sought by all the world, to-day we saw the D. D. S. virtually disestablished in Europe, and in some cases made even a term of reproach. And all this had been brought about—not by the profession in America—but by the American colleges themselves. American dentists had felt the blush of shame mount their cheeks, when they were constantly reminded that the degree of D. D. S., the distinctive mark of an American dentist, had been openly hawked about Europe *for sale.* The diplomas of the Wisconsin Dental College were offered, even in this country, to any one who would pay for them. And Wisconsin was a regularly chartered school, that conferred degrees "by merit," and not upon any time basis. This was the plea—that of recognizing merit—under which all the diploma rascality of America, whether in medical or dental colleges, had been committed. The plea was a very plausible one, but it could be easily seen how it might be, and was, abused.

But it was not the Wisconsin College alone that was the offender. Reputable colleges had engaged in the business of conferring diplomas upon foreign students, after an attendance that was but a mere farce and sham; upon students who were not even acquainted with the English language, and hence could not comprehend the few lectures that they did attend. Seeing these things, the Dental Society of the State of New York thought it was time that it should put forth the power granted it by the Legislature, and call the colleges to an account, as they were accountable to no other power. Hence the passage of this resolution, which requested the Baltimore College to show cause. He regretted that in so doing they should not have given opportunity for immediate answer, but he thought the action was called for. Now, as Baltimore had receded from the ground it had occupied, and would consider itself bound by the agreement of the National Board, he moved that the Dental Society of the State of New York express its entire confidence in the Baltimore Dental College.

*Dr. N. W. Kingsley*—Inquired if the mover desired to base the resolution upon the fact of the abandonment by the College of the plan of granting degrees upon a mere examination.

*Dr. Barrett*—Said that he did not wish to embody this in the resolution, although of course it was taken into consideration. He did not believe that the Dental Society of the State of New York would ever express its confidence in any college that granted its diplomas *in course*, without a regular and due attendance upon lectures, nor that it would sanction the conferring of degrees upon a mere examination. There was a difference of opinion among its members concerning the propriety of granting the degree of Master of Dental Surgery, but there was this much to be said for it: it was not a degree in course, and did not pretend to be proof that its possessor had pursued any prescribed curriculum of study. It was not a degree of the schools at all, nor did it pretend to be. It was the child of the late Dr. Amos Wescott, a well known teacher, and when he recommended it no one questioned its propriety, and so it was incorporated in the State law, and the State Society would stand by and support it until such time as we could harmoniously agree upon its abolishment. No member would become factional over it, but we would all act together for what we thought best. There were differences of opinion about the degree, but every one might rest assured that it would be supported loyally by every member, so long as it was a part of the law.

A number of amendments and substitutes were offered for Dr.

Barrett's motion, but all were voted down, and the original motion was finally put and carried.

Prof. Follett, of the Boston Dental College, presented the claims of that institution for recognition by the State Society, that its diplomas might be legalized in this State. He stated the only basis upon which degrees were granted, and read a recommendatory letter from Prof. Chandler, of the Harvard Dental School.

Upon motion the matter was referred to the Board of Censors, and they subsequently recommended that the name of the College be placed upon the list, which was accordingly done.

The committee upon the Whitney Memorial Prize Essay fund reported, recommending that the annual cash prize be awarded to Dr W. H. Atkinson, for a paper entitled "The Reproduction of Tissue by Sponge Grafting."

On motion the Society adjourned until the evening session.

(TO BE CONTINUED.)

